# Weak global warming mitigation by reducing black carbon emissions

**DOI:** 10.1038/s41598-019-41181-6

**Published:** 2019-03-14

**Authors:** Toshihiko Takemura, Kentaroh Suzuki

**Affiliations:** 1 Research Institute for Applied Mechanics, Kyushu University, Fukuoka, Japan; 2 Atmosphere and Ocean Research Institute, University of Tokyo, Kashiwa, Japan

## Abstract

Reducing black carbon (BC), i.e. soot, in the atmosphere is a potential mitigation measure for climate change before revealing the effect of reducing anthropogenic carbon dioxide (CO_2_) because BC with shorter lifetime than CO_2_ absorbs solar and infrared radiation. BC has a strong positive radiative forcing in the atmosphere, as indicated in many previous studies. Here, we show that the decline in surface air temperatures with reduced BC emissions is weaker than would be expected from the magnitude of its instantaneous radiative forcing at the top of the atmosphere (TOA). Climate simulations show that the global mean change in surface air temperature per unit of instantaneous radiative forcing of BC at the TOA is about one-eighth that of sulphate aerosols, which cool the climate through scattering solar radiation, without absorption. This is attributed to the positive radiation budget of BC being largely compensated for by rapid atmospheric adjustment, whereas the radiative imbalance due to sulphate aerosols drives a slow response of climate over a long timescale. Regional climate responses to short-lived species are shown to exhibit even more complex characteristics due to their heterogeneous spatial distributions, requiring further analysis in future studies.

## Introduction

The Paris Agreement is intended to keep increases in the global mean surface air temperature to below 2 °C relative to preindustrial levels and to make efforts toward limiting that increase to 1.5 °C. Although reducing greenhouse gas emissions is the predominant mitigating measure in achieving that goal, the effect of this measure on global mean surface air temperatures will not be observed over the course of the next few decades, because after being emitted carbon dioxide (CO_2_) has an atmospheric lifetime over a time scale of a century. It has been suggested that reducing other greenhouse species with shorter atmospheric lifetimes than CO_2_ might be a way to mitigate global warming before the effect of CO_2_ reduction becomes apparent. Most short-lived greenhouse species are harmful to human health, so reducing them is also crucial in the context of air quality and would result in a co-beneficial effect. Such pollutants are referred to by the Climate and Clean Air Coalition (CCAC)^1^ as short-lived climate pollutants (SLCPs) and consist of black carbon (BC), methane (CH_4_), tropospheric ozone (O_3_), and hydrofluorocarbons (HFCs).

Two significant issues emerge when attempting to achieve co-beneficial effects on climate change and air quality via reducing pollutant emissions. One is that some air pollutants, such as sulphate and nitrate aerosols, cool the atmosphere; simply reducing them would accelerate global warming. This implies that the overall impact of air pollutants in both warming and cooling the climate need to be assessed when seeking a co-beneficial effect. The Intergovernmental Panel on Climate Change (IPCC) considers them as short-lived climate forcers (SLCFs), regardless of whether the radiative forcing, an imbalance of the energy budget affected by a change in their concentration, is positive or negative. An IPCC Assessment Report^2^ indicated that global mean radiative forcing due to total anthropogenic aerosols is negative, meaning that a reduction in their emissions will accelerate global warming. To mitigate air pollution and acid rain, developed countries have primarily reduced emissions of sulphur dioxide (SO_2_), a primary precursor of sulphate aerosols.

The other issue is that although BC is associated with a strong positive radiative forcing effect, recent studies have found that the sensitivity of surface air temperatures to BC tends to be weaker than expected^3–6^. Past and future climate simulations used in the IPCC Assessment Reports^7,8^ all included the effects of all major climate forcing agents, however their individual effects on meteorological parameters (e.g. temperature and precipitation) were not assessed. In an attempt to fill this gap, the Precipitation Driver Response Model Intercomparison Project (PDRMIP) recently analysed responses of precipitation and temperature to individual perturbation of some climate forcing agents using both atmosphere-only and ocean-coupled general circulation models^5^. The PDRMIP protocol included simulations of climate change with extreme perturbations of sulphate and BC aerosol concentrations or emissions. It predicted that increasing present-day concentrations or emissions of BC by even a factor of 10 would result in only small changes in surface air temperatures due to the dominant rapid adjustment of the climate system, including an increase in low-level clouds^6^. However, assessment of perturbation of each climate forcing agent individually, particularly with such an unrealistic magnitude, is not sufficient to assess the climate response fully over a range of perturbations, including realistic perturbations. This is essential when making scientifically based decisions about potential measures to mitigate SLCPs/SLCFs, as well as about well-mixed greenhouse gases, such as CO_2_.

Here, we investigate the quantitative trend in surface air temperatures with multiple perturbed emissions of BC and SO_2_ in the realistic range (zero to twice) relative to the present conditions using a general circulation model coupled with aerosol processes, known as MIROC-SPRINTARS^9–11^. Contrary to the previous studies that took multi-model ensembles for each experiment with only one perturbation per species^3–6^, an advantage of this single-model approach is that the results are not contaminated with differences in physical representations that vary across models, enabling consistent investigation of the relationship between the energy budget perturbation and the climate response. The MIROC-SPRINTARS calculates changes in meteorological parameters by aerosol-radiation interactions in which aerosols scatter and absorb solar and thermal radiation, and aerosol-cloud interactions in which aerosols alter cloud microphysical properties and affect the radiation budget by acting as cloud condensation and ice nuclei. Here, two simulations are executed to distinguish climate changes due to rapid adjustment and slow response: one with prescribed sea surface temperatures and sea ice and the other coupled with an ocean general circulation model (OGCM) (see Methods).

## Results

**Figure 1:**
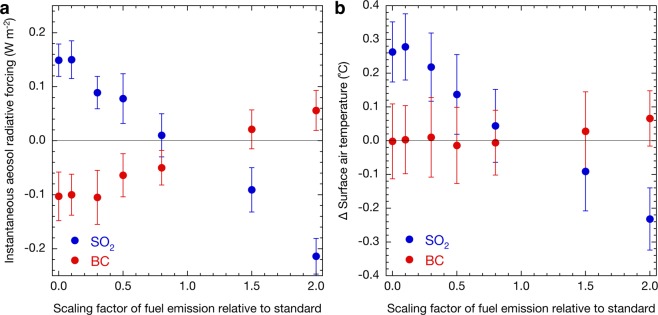
Sensitivities to changes in SO_2_ and BC emissions. Global mean instantaneous aerosol radiative forcing (**a**) and change in the mean surface air temperature (**b**) with emission perturbations of SO_2_ (blue) and BC (red) from fuel sources relative to the present emissions simulated by MIROC-SPRINTARS. Error bars represent one standard deviation in annual mean values. Changes in surface air temperatures are from the simulations coupled with OGCM.

Figure 1 shows how instantaneous radiative forcing at the top of the atmosphere (TOA) and surface air temperatures change with perturbed emissions of SO_2_ and BC from fuel sources. Although the global mean instantaneous radiative forcing values at the TOA simulated in this study (Fig. 1a) are somewhat lower than the maximum likelihoods shown in the latest IPCC Assessment Report^2^, they are within the uncertainty range. Assessment of the uncertainty should be extended to multi-model comparisons, such as the Aerosol Comparisons between Observations and Models (AeroCom)^12^, in which the MIROC-SPRINTARS is participating. It is important when using a single-model approach to indicate almost linear trends in the instantaneous radiative forcing at the TOA with perturbed emissions for both sulphate and BC aerosols (Fig. 1a).

**Figure 2:**
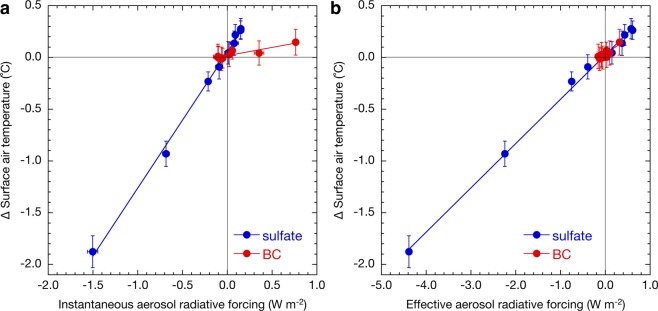
Relationship between aerosol (**a**) instantaneous or (**b**) effective radiative forcing and changes in surface air temperatures. Each point shows individual simulated results with various scaling factors relative to present emission amounts, as shown in Methods, for sulphate (blue) and BC (red) aerosols by MIROC-SPRINTARS. Error bars represent one standard deviation in annual mean values. The correlation coefficients for the regression lines are 1.00 and 0.91 in (**a**) and 1.00 and 0.88 in (**b**) for sulphate and BC aerosols, respectively.

Changes in surface air temperatures are traditionally and conveniently estimated from instantaneous radiative forcing, which is related to climate sensitivity. Figure 1a shows that if the climate sensitivity for BC is equal to that for sulphate aerosols, the mean change in the surface air temperature due to reducing BC emissions is expected to be approximately two-thirds of the change if SO_2_ emissions increase. However, Fig. 1b shows that the simulated decrease in the global mean surface air temperature when reducing BC emissions is much smaller than expected, to the extent that for a realistic change in BC emissions the trend is lost in the interannual variability. It is notable that the relationship between the instantaneous radiative forcing and the change in the surface air temperature is almost linear for both sulphate and BC aerosols (Fig. 2a). The climate sensitivity parameter (i.e. the gradient of plots shown in Fig. 2a) estimated by MIROC-SPRINTARS for sulphate aerosols (1.3 °C W^−1^ m^2^) is eight times larger than that for BC (0.16 °C W^−1^ m^2^). This implies that instantaneous radiative forcing, used when assessing the effect on climate of BC, with climate sensitivity implicitly assumed to be the same as that of sulphate, does not serve as a good predictor of the temperature response.

## Discussion

The reason for the weak response of the mean surface air temperature to BC perturbation is that instantaneous radiative forcing is largely compensated for by rapid adjustment (Table 1). The compensation consists of changes in the clear-sky radiative flux and cloud radiative effect at the TOA. Under the condition of reducing BC emission which weakens the atmospheric radiative heating associated with the instantaneous radiative forcing, the clear-sky radiative cooling weakens due to a decrease in water vapor. Reducing BC emission also makes atmosphere unstable, leading to decreases in low-clouds and increases in middle- and high-clouds, which results in a decrease in outgoing radiation. Both changes in the clear-sky radiative flux and cloud radiative forcing counteract the instantaneous radiative forcing in the BC case. As a result, only a fraction of the perturbed energy is left to drive the slow response accompanied by temperature change (Table 1). In contrast, the instantaneous radiative forcing of sulphate is almost the same between the TOA and surface, causing little radiative heating/cooling in the atmosphere. Then the clear-sky radiative flux is unchanged. On the other hand, an imbalance in the radiation budget induced by sulphate is enhanced by the cloud radiative effect, which primarily consists of the aerosol-cloud interaction for water clouds (Table 1). The radiative forcing of sulphate from fuel sources due to the aerosol-cloud interaction is estimated to be −0.36 W m^−2^ in this study, which is comparable to −0.45 W m^−2^ by all anthropogenic aerosols in the latest IPCC Assessment Report^2^.

**Table 1:** Changes in the normalised global mean energy budget at the top of the atmosphere when reducing SO_2_ and BC emissions.

	Instantaneous radiative forcing	Rapid adjustment	Slow response
SO_2_ (sulfate)	+1.0	+2.3	−2.4
BC	−1.0	+0.6	+0.4

Values are expressed in W m^−2^. Global mean values are normalised to instantaneous radiative forcing.

The remarkable difference in TOA energy balance change between BC and sulphate arises from different vertical structures of the instantaneous radiative forcing, which induce rapid adjustments counteracting or enhancing the forcing through different energy redistributions into atmosphere and surface^13^. This involves opposite effects of cloud responses that buffer and enhance the instantaneous radiative forcing for BC and sulphate cases, respectively. In the latter, the TOA energy imbalance is swelled by the radiative forcing due to the aerosol-cloud interaction and is not fully cancelled by slow response (Table 1) implying energy exchange with ocean. These different characteristics of energy budget change is a basic mechanism responsible for different responses of the surface air temperature to the instantaneous radiative forcing. In the context of recent PDRMIP intercomparison, the temperature sensitivity to 5 times SO_2_ emissions or sulfate concentrations relative to the present condition simulated by MIROC-SPRINTARS is within a range of uncertainty among multiple models^5^. The present results quantify sensitivities of the surface air temperature to varying emissions of BC and sulphate over realistic ranges to particularly highlight the weaker temperature response than would be expected from the BC-induced instantaneous radiative forcings at TOA. This is explained by the energy budget change that occurs not only at the TOA but also in the atmosphere and at the surface involving the latent and sensible heat fluxes^13^, which also cause changes to precipitation as well as temperature.

The present results, together with recent multi-model findings^4–6^, require re-investigation of the CCAC activity that is based on an integrated scientific assessment by the United Nations Environment Programme (UNEP). In the assessment report^14^, the temperature sensitivities to all the aerosol species per instantaneous radiative forcing are assumed to be the same as that of CO_2_. However, this assumption, with the weaker temperature sensitivity to BC emission change not considered, tends to overestimate the expected mitigation of global warming by reducing BC emission. Given that BC reduction is mandatory for avoiding an impact on human health, this study proposes that long-lived greenhouse gases and other SLCPs should be reduced more firmly for mitigating global warming.

The discussion above underscores an importance of considering rapid adjustment that substantially modulates the energy budget response to BC emission change. This is facilitated by using the effective radiative forcing, which includes the instantaneous radiative forcing and the rapid adjustment as defined in the fifth assessment report of the IPCC^2^ and shown to be a better predictor of the temperature change. Indeed, the climate sensitivity parameters based on the effective radiative forcing simulated by MIROC-SPRINTARS are 0.43 and 0.31 °C W^−1^ m^2^ for sulphate and BC aerosols respectively, which shows a smaller discrepancy than that for instantaneous radiative forcing (Fig. 2b).

**Figure 3:**
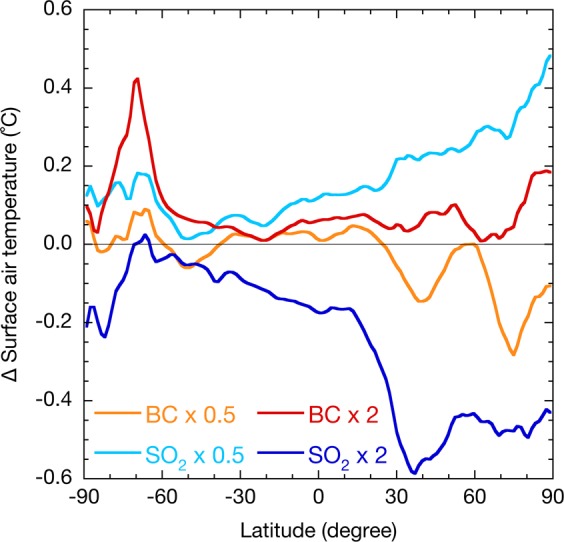
Latitudinal distributions of changes in surface air temperatures with aerosol emissions. The annual mean surface air temperatures with scaling factors of 0.5 and 2 for SO_2_ (blue) and BC (red) relative to the present emission amounts simulated by the MIROC-SPRINTARS.

These findings imply that reducing atmospheric BC concentrations may not be effective for the decline in global surface air temperatures, although such reduction is crucial for air quality. Therefore, reducing emissions of well-mixed greenhouse gases, i.e. CO_2_, CH_4_, nitrous oxide (N_2_O), and halocarbons, as well as other SLCPs (CH_4_, tropospheric O_3_, and HFCs), is an essential mitigation measure in the pursuit of the goals within the Paris Agreement. However, heterogeneous spatial distributions of SLCFs can result in large changes in surface air temperatures in the mid- and high-latitudes of the Northern Hemisphere (Fig. 3). Detailed analysis will be needed in future studies to investigate the effects of SLCFs on regional climate change.

## Methods

A global ocean-atmosphere general circulation model, MIROC^11^, and an aerosol processes model, SPRINTARS^9,10^, were used in this study. The coupled MIROC-SPRINTARS models predicted wind, temperature, surface pressure, specific humidity, mass mixing ratios of cloud water/ice and major aerosol components (sulphate, BC, organic matter (OM), soil dust, and sea salt and precursor gases of sulphate including SO_2_ and dimethyl sulphide) and precipitation flux, etc. These meteorological parameters in MIROC-SPRINTARS change via aerosol-radiation and aerosol-cloud interactions. The simulated transport processes of aerosols and their precursors include emission, advection, diffusion, chemical reactions of sulphur, wet deposition, and dry deposition. Emission fluxes from fuel and biomass burning sources of SO_2_, BC, and OM were acquired from the Emissions Database for Global Atmospheric Research and Task Force on Hemispheric Transport of Air Pollution (EDGAR-HTAP)^15^ and Global Fire Emissions Database (GFED)^16^. Emissions from natural sources, such as soil dust and sea salt, were calculated from the internal parameters of the model. A change in the snow albedo due to deposition of BC and soil dust was parameterised. A detailed model description of MIROC-SPRINTARS is provided in a previous study^17^.

Sensitivity tests were performed with various scaling factors (i.e. 0, 0.1, 0.3, 0.5, 0.8, 1.5, 2, 5, and 10), which are globally homogeneous relative to the standard emission amount of SO_2_ and BC from fuel sources, i.e. the EDGAR-HTAP database. The emission scaling factors were only 5 and 10 times for SO_2_ and BC, respectively, in the PDRMIP model intercomparison experiments^18^. To understand the climate effects of sulphate and BC aerosols due to the total response to rapid adjustment, both the simulations with prescribed sea surface temperatures (SSTs) and sea ice^19^ and with an ocean general circulation model (OGCM)^11^, were executed for each of the perturbed emissions. The results of the simulations with prescribed SSTs and sea ice were defined to represent the rapid adjustment only, and those with the OGCM included both rapid adjustment and slow responses^18^. The slow response was estimated as the difference between the results from the two simulations. The instantaneous radiative forcing was calculated by a ‘double call’ in which the atmospheric radiation process in the climate model is called twice with and without the aerosol-radiation interaction in a time step using the simulations with prescribed SSTs and sea ice. The simulations are integrated for 15 and 100 years in the prescribed SSTs and sea ice and ocean-coupled experiments, respectively, and the simulated results were analysed for the last 10 and 50 years, respectively. The horizontal resolution of all simulations was T85 (approximately 1.4° by 1.4° in longitude and latitude) and the vertical resolution was 40 layers with the hybrid sigma-pressure coordinate.
